# Case Report: A Novel Mutation Identified in *CHST14* Gene in a Fetus With Structural Abnormalities

**DOI:** 10.3389/fgene.2022.853907

**Published:** 2022-04-08

**Authors:** Yuan-Yuan Zhou, Yu-Fang Du, Qing Lu, Xiu-Zhang Zhai, Ming-Fang Shi, Dan-Yun Chen, Sun-Rong Liu, Ying Zhong

**Affiliations:** Department of Clinical Laboratory, The Third Affiliated Hospital of Guangxi Medical University, Nanning, China

**Keywords:** whole-exome sequencing, prenatal diagnosis, *CHST14*, Ehlers–Danlos syndrome, structural abnormalities

## Abstract

**Background:** Musculocontractural Ehlers–Danlos syndrome (mcEDS) is a rare heritable connective tissue disease with various symptoms. The diagnosis of mcEDS is difficult because of the large overlap of clinical symptoms between different EDS subtypes.

**Methods:** We performed karyotype analysis, gene copy number variation detection, whole-exome sequencing, and Sanger sequencing to reveal the underlying genetic etiology of a fetus with structural abnormalities in feet and kidneys.

**Results:** A likely pathogenic mutation [NM_130468.3 c.958C>T (p.Arg320*)] and an uncertain significance mutation [NM_130468.3 c.896A>G (p.Tyr299Cys)] were identified in the carbohydrate sulfotransferase 14 (*CHST14*) gene by whole-exome sequencing and validated by Sanger sequencing.

**Conclusion:** The two identified mutations appear highly likely to be the genetic causes of the fetal structural abnormalities.

## 1 Introduction

Fetal structural abnormalities emerge in approximately 3.0% of pregnancies, which can be related to all types of genetic variants (Persson et al., 2017; Lord et al., 2019). Karyotyping and chromosomal microarray analysis are recommended as the preferred diagnostic methods for fetal structural abnormalities (International Society for Prenatal Diagnosis, 2018). However, more than 60% of fetal structural abnormalities cannot be explained by chromosomal karyotyping and microarray analysis (Wapner et al., 2012). Recently, whole-exome sequencing (WES) has been confirmed to be a valuable diagnostic approach for explicating the underlying genetic etiology for many likely Mendelian disorders (Petrovski et al., 2019).

The Ehlers–Danlos syndromes (EDS) are a group of heritable connective tissue diseases involving at least 17 genes and 13 subtypes, with various symptoms, characteristically joint hypermobility, skin hyperextensibility, and tissue fragility (Malfait et al., 2017). Musculocontractural Ehlers–Danlos syndrome (mcEDS) is a subtype of EDS caused by homozygous or compound heterozygous mutations of the carbohydrate sulfotransferase 14 (*CHST14*) or dermatan sulfate epimerase gene (Malfait et al., 2010). Three major clinical criteria are defined for the diagnosis of mcEDS, including 1) congenital multiple contractures, typically adduction-flexion contractures, and/or talipes equinovarus (clubfoot); 2) characteristic craniofacial features; and 3) characteristic cutaneous features, for example, skin hyperextensibility, easy bruisability, and skin fragility (Malfait et al., 2017).

The diagnosis of EDS once mainly relied on clinical features (Beighton et al., 1988). Then, in 1997, the biochemical and molecular bases were required to classify EDS (Beighton et al., 1998). A molecular confirmation is very important for the diagnosis and counseling in view of the overlap of clinical symptoms between different EDS subtypes. Recently, it has been recommended that molecular detection should base on next-generation sequencing technologies, such as copy number variation (CNV) detection, WES, and whole-genome sequencing (WGS) (Malfait et al., 2017). Prenatal diagnosis of mcEDS is more difficult than postnatal diagnosis, as the craniofacial and cutaneous features have not been represented completely. Up till now, prenatal mcEDS has not yet been reported. We herein introduce an mcEDS case diagnosed by prenatal WES and Sanger sequencing.

## 2 Materials and Methods

### 2.1 Case Information

A 34-year-old woman with 22-week gestation visited the Department of Genetic Counseling of the Third Affiliated Hospital of Guangxi Medical University (Nanning, China) for genetic counseling on the fetal abnormalities revealed by ultrasound examination. The ultrasound report showed adduction flexion (Supplementary Figure S1) in the fetal feet. The renal pelvis in both kidneys was separated, the left test was as large as 5.5 mm, and the right test was 5.2 mm. The estimated weight of the fetus was about 539 g. The fetal biparietal diameter, head circumference, abdominal circumference, transverse diameter of cerebellum, length of the humerus, and length of the femur were 53, 198, 182, 24, 39, and 40 mm, respectively. The woman and her husband were both in good health conditions. Family history of genetic diseases and consanguineous marriage were denied by the couple.

### 2.2 Amniotic Fluid Cell Karyotype Analysis

A total of 20 ml amniotic fluid was obtained under the guidance of ultrasound by an experienced obstetrician. After that, 15 ml of amniotic fluid was transferred into two cell culture bottles and then placed in an incubator with 37°C and 5% CO_2_ for a week. Chromosomes are prepared according to a routine chromosomal collection process, and at least 40 meta-phase cells were analyzed by two experienced technicians using the ZEISS meta-system (CARL ZEISS AG, Jena, Germany).

### 2.3 DNA Extraction

Fetal DNA was extracted from 5 ml amniotic fluid, and biological parental DNA was extracted from corresponding venous blood using the introduction of the QIAmp DNA extraction Kit (QIAGEN, Dusseldorf, Germany). All DNA was stored at −80°C after extraction.

### 2.4 Copy Number Variation Sequencing

Library construction was performed through a series of experiments, including DNA fragmentation, label ligation, pre-PCR purification, PCR, and post-PCR purification, according to the standard operation procedures (CapitalBio, Beijing, China). CNV-seq was performed using the bio-electronseq 400 (CapitalBio, Beijing, China) and the life ion torrent platform (CapitalBio, Beijing, China). The lower detective limits of CNV-seq are 100 kb for micro-deletion and micro-duplication and 10% for mosaicism.

### 2.5 Whole-Exome Sequencing and Sanger Sequencing

Library preparation was carried out according to the standard procedure (Basic Graphics Interface (BGI), Shenzhen, China). BGI V4 chip was used to capture and enrich the exome of target genes. Mgiseq-2000 sequencing platform (BGI) was employed to detect gene variations. The sequencing reads were compared with the genome UCSC hg19 by the Burrows–Wheeler Aligner. The Genome Analysis Toolkit (Broad Institute, Cambridge, MA, United States) was used to detect single nucleotide variations, basal insertion, and genotype. EXOME DEPTH was used to test copy number variation at the exome level. Sanger sequencing was performed to validate any identified mutation. The pathogenicity was evaluated according to the guidelines of the American College of Medical Genetics and Genomics (ACMG) (Richards et al., 2015) and analyzed in three databases: SIFT, PolyPhen, and MutationTaster.

## 3 Results

### 3.1 Results of Chromosomal Karyotyping and CNV Sequencing

The fetal chromosomal karyotype was normal, and no known pathogenic micro-deletion (>100 kb), pathogenic micro-duplication (>100 kb), mosaicism (>10%), or aneuploidy was detected in the fetus.

### 3.2 Whole-Exome Sequencing Analysis

A total of 405 variants (Supplementary Table S1) were filtered out by a filtering process (Supplementary Figure S2). Two mutations identified in the *CHST14* gene of the fetus were considered of clinical significance. According to the ACMG guidelines [NM_130468.3 c.958C>T (p.Arg320*)] was classified as a likely pathogenic variant and [NM_130468.3 c.896A>G (p.Tyr299Cys)] was uncertain significance. Meanwhile, the likely pathogenic variant was also identified in the mother, and the uncertain significance variant was also identified in the father. Predicted pathogenicity is shown in Table 1.

**TABLE 1:** Two mutations identified in the *CHST14* gene.

Cytogenetic location/gene subregion	Variants (protein) [RefSeq ID]	Inheritance/zygosity	Detection of family members	Disease association(s) [MIM #]	Pathogenicity (ACMG guidelines/SIFT/PolyPhen/MutationTaster)
chr15:4076-4370/EX1E	c.958C>T (p.Arg320*) [NM_130468.3]	AR/het	Mother (het)	mcEDS [601776]	Likely pathogenic/—/—/disease-causing
chr15:4076-4308/EX1E	c.896A>G (p.Tyr299Cys) [NM_130468.3]	AR/het	Father (het)	mcEDS [601776]	Uncertain significance/damaging/probably damaging/disease-causing

AR, autosomal recessive; EX1E, exome 1E region; het, heterozygous; mcEDS, musculocontractural Ehlers–Danlos syndrome.

### 3.3 Sanger Sequencing Validation

**FIGURE 1:**
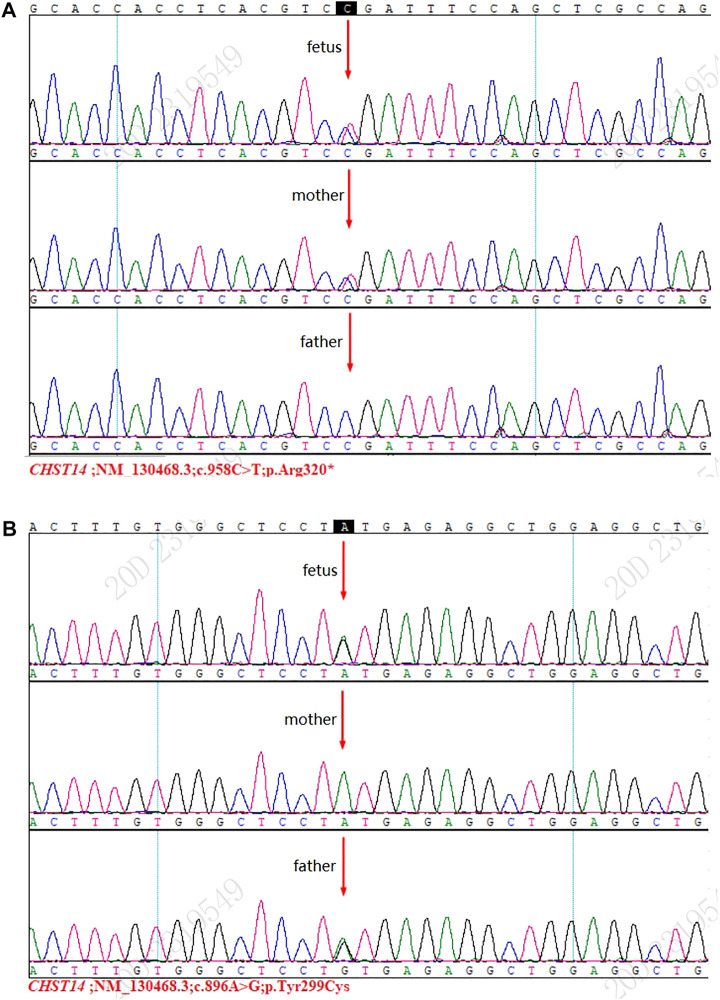
Results of Sanger sequencing. **(A)** Mutation [NM_130468.3 c.958C>T (p.Arg320*)] was detected in the fetus and the mother. **(B)** Mutation [NM_130468.3 c.896A>G (p.Tyr299Cys)] was detected in the fetus and the father.

Sanger sequencing detected two mutations [NM_130468.3 c.958C>T (p.Arg320 *)] and [NM_130468.3 c. 896A>G (p.Tyr299Cys)], and these results were consistent with those of WES (Figures 1A,B).

## 4 Discussion

Next-generation sequencing such as CNV-seq, WES, and WGS has been widely used in clinical practice in the last decade. It has been reported that WES-trio achieved a diagnostic rate of 40% in diagnosing genetic disorders, which was almost as high as that of WGS-trio (42%) (Lord et al., 2019). Seven genes implicating stillbirth were identified by WES, with a detection rate of 6.1% (Stanley et al., 2020). Therefore, WES has recently been recommended to be used in prenatal clinical practice to uncover the underlying genetic causes of fetal structural anomalies while abnormal karyotype and pathogenic CNV had been excluded (Petrovski et al., 2019).

*CHST14* gene is located in number 15 chromosome (15q15.1), involving only one exon and encoding N-acetylgalactosamine 4-O-sulfotransferase 1 (D4ST1), which plays an essential role in the biosynthesis of proteoglycans (CHST14 carbohydrate sulfotransferase 14 [Homo sapiens (human)] - Gene - NCBI (nih.gov)). Proteoglycans are abundant in the extracellular matrix and important in a wide range of physiological functions, such as interacting with collagen (Malfait et al., 2020). Pathogenetic mutations in the *CHST14* gene result in deficiency of D4ST1, which consequently leads to the decrease of proteoglycans and further abnormal regulation of collagen fibrils assembly and finally gives rise to the mcEDS (Dundar et al., 2009; Malfait et al., 2020).

To our best knowledge, at least 26 variants of the *CHST14* gene have been reported. However, no apparent relationship between genotype and phenotype is noted (Minatogawa et al., 2021). Dundar et al. (2009) discovered a 1 bp deletion (c.145_146 delG), a missense mutation (c.638G>C), and a complex allele (c.404C>G; 410T>A) in *CHST14* in Australian Turks with thumb-clubfoot Syndrome. Miyake et al. (2010) reported four mutations (c.842C>T p.P281L, c.866G>C p.C289S, c.878A>G p.Y293C, c.205A>T p.K69*) of the *CHST14* gene in six Japanese patients with Kosho type EDS. In fact, thumb-clubfoot syndrome and Kosho type EDS have a common clinical condition, so they are termed mcEDS (Malfait et al., 2010).

In this study, novel and reported mutations in *CHST14* were detected in a fetus with adduction flexion in the feet and renal pelvis in the kidneys. A likely pathogenic mutation [NM_130468.3 c.958C>T (p.Arg320*)], which was also found in the mother, had been reported previously by Minatogawa et al. (2021) in a study involving 66 mcEDS patients. A novel variation [NM_130468.3 c.896A>G (p.Tyr299Cys)], which was also found in the father, was evaluated as an uncertain significance mutation according to the ACMG guidelines. However, it was predicted to be a disease-causing or probably damaging mutation in *in silico* analyses. The two detected variants are located in the middle of the sulfotransferase domain and presumably result in partial or complete loss of function of D4ST1 (Minatogawa et al., 2021).

The couple decided to terminate this pregnancy after genetic counseling. Clubfeet (Supplementary Figure S3) were confirmed by autopsy, and this was consistent with the ultrasound results. The autopsy record about kidneys was unknown. Clubfoot was one of the three major criteria for diagnosing mcEDS and 95% (59/62) mcEDS patients developed clubfoot, while renal structural abnormalities were not observed (Minatogawa et al., 2021). By considering the clinical symptom (clubfeet) and the molecular detective results, an alternative diagnosis of mcEDS was made to the fetus. It was clear that the fetus inherited the two mutations from both parents and became a carrier of compound heterozygous mutations of the *CHST14* gene. It appears highly likely that the structural abnormalities, especially clubfeet, are caused by the two mutations. However, further functional studies, such as cell experiments, are needed to support the assumption.

In conclusion, we identified a novel mutation [NM_130468.3 c.896A>G (p.Tyr299Cys)] and a reported likely pathogenic mutation [NM_130468.3 c.958C>T (p.Arg320*)] in the *CHST14* gene of by WES prenatally, which can perhaps be claimed as the potential genetic etiology of the fetal structural abnormalities.

## Data Availability

The datasets for this article are not publicly available due to concerns regarding participant/patient anonymity. Requests to access the datasets should be directed to the corresponding author.

## Supplementary Material

Supplementary Figure S1 Adduction-flexion revealed by ultrasound.

Supplementary Figure S2 Filtering process of WES data.

Supplementary Figure S3 Clubfeet in the fetus.
