# Supplementary figures and images for: Case Report: A Novel Mutation Identified in CHST14 Gene in a Fetus With Structural Abnormalities
Source: Front Genet. 2022 Apr 8;13:853907. doi: 10.3389/fgene.2022.853907 (PMC9024400; doi:10.3389/fgene.2022.853907)

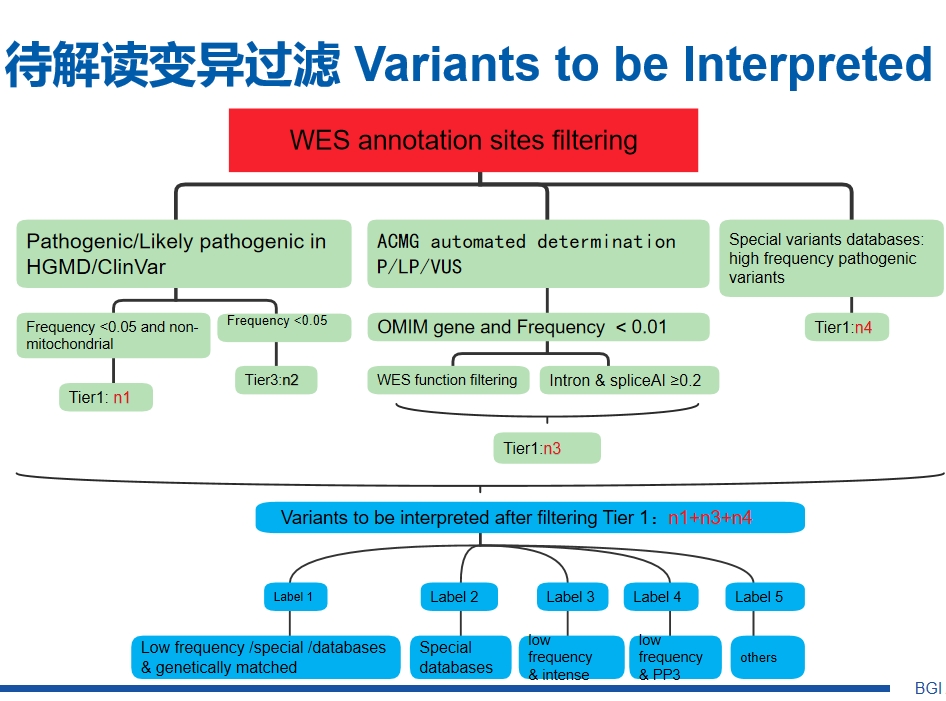

Supplement: Supplementary file 1 [file Image3.jpg]

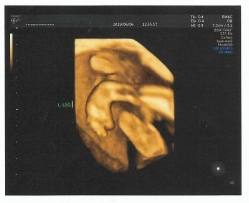

Supplement: Supplementary file 2 [file Image2.jpg]

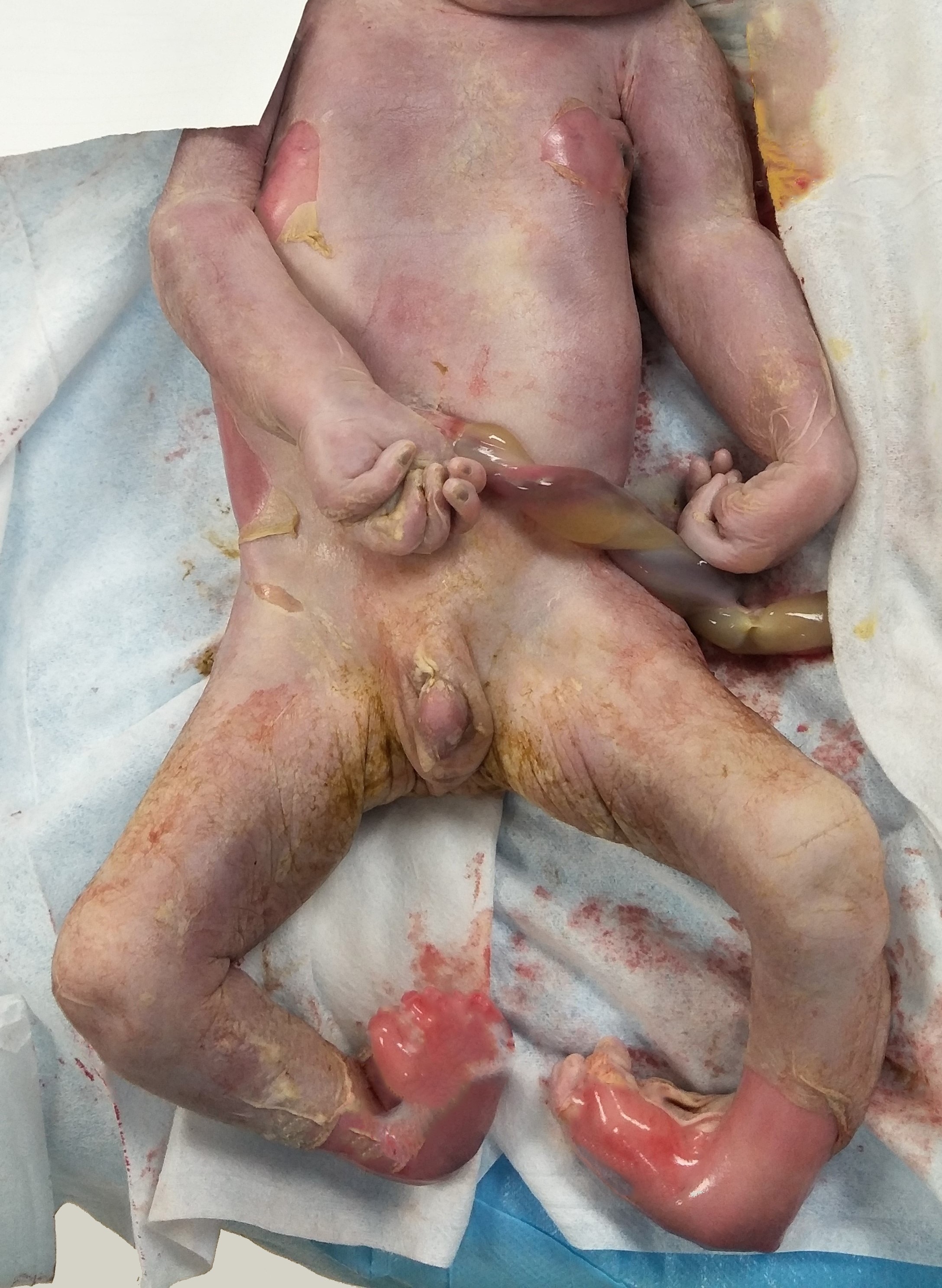

Supplement: Supplementary file 4 [file Image1.jpg]
